# Concern for the Health of Ethiopia's Elderly Population: A Call for a Comprehensive Geriatric Medicine Strategy

**DOI:** 10.4314/ejhs.v35i2.1

**Published:** 2025-03

**Authors:** Woldesenbet Waganew Dode, Sena Dhugasa Teso

**Affiliations:** 1 St. Paul Hospital Millenium Medical College, Department of emergency and critical care, Addis Ababa, Ethiopia

Aging population is a human success story, reflects the progress made in economic and social development, public health, and medicine, as well as their role in preventing injury, controlling disease, and lowering the risk of premature death (1). Those aged 60 years and above are considered as older in Sub-Saharan Africa (2). The percentage of individuals aged 60 and above is predicted to rise remarkably in the coming decades, as per World Health Organization. Ethiopia, an older and historically rich Sub-Saharan African nation with a population of over 115 million, is experiencing a demographic shift characterized by an increasing elderly population (3). By 2050, Ethiopia's older adult population is predicted to more than double from 5.1% in 2010 to 10.3% (4). Therefore, this demographic change, demands great attention of policy and the health system that can fit this transition, particularly in geriatric medicine, which focuses on the health care of older adults (5). Despite Ethiopia's demographic shift toward a larger percentage of the elderly population, policies and programs are not implemented to address this (3, 4).

Serious consequences follow from inadequate geriatric healthcare. Elderly communities commonly having chronic illnesses, such as diabetes, hypertension, and arthritis, addressing both psychosocial and medical requirements in comprehensive manner is essential. Strong geriatric care strategy shall be in place in health care system to deliver suitable service, without which there might be high morbidity and mortality among senior citizens. Furthermore, existing health inequities may be made worse by a shortage of qualified healthcare providers with geriatric medicine specialization (1,2, 4-5).

Developing comprehensive strategy that includes several crucial elements is required for Ethiopia to overcome to address the difficulties in geriatric care provision. First and foremost, national policy, that give due attention for geriatric healthcare must be established. These policies should include financing for research and the creation of age-friendly health systems. The need for institutionalization will decrease if community-based healthcare efforts are supported, allowing older persons to receive care in their homes or local communities. Furthermore, funding specialized training programs for geriatric healthcare workers is crucial to ensuring that they are unique needs of senior individuals. Additionally, encouraging equipped to manage the cooperation between other fields, such as social services, healthcare, and community organizations, will enable comprehensive treatment that attends to both social and medical needs. Finally, launching campaigns to educate families and communities about the importance of geriatric care and the available resources will help raise awareness and support for this vital area of healthcare (1,4-5).

As Ethiopia's elderly population continues to grow, it is imperative that the nation adopts a comprehensive strategy for geriatric medicine. By addressing the unique healthcare needs of older adults through targeted training, policy development, community-based initiatives, and public awareness efforts, Ethiopia can improve health outcomes for its aging population. If these steps are not taken, the consequences will be far-reaching, affecting not only the elderly but also families and society at large. The time for action is now; a proactive approach will ensure that Ethiopia's healthcare system is prepared to meet the challenges posed by an aging population.
